# Whole-Body Pharmacokinetics of Lipid, mRNA and Translated Protein Following Intravenous Administration of Spike Protein Expressing mRNA-LNP in Mice

**DOI:** 10.1007/s11095-026-04086-4

**Published:** 2026-04-02

**Authors:** Mokshada Kumar, Shrusti Tiwari, Aneesh Rajwade, Rutuja Kulkarni, Arhan Patel, Dhaval K. Shah

**Affiliations:** Department of Pharmaceutical Sciences, School of Pharmacy and Pharmaceutical Sciences, The State University of New York at Buffalo, 455 Pharmacy Building, Buffalo, NY 14214-8033 USA

**Keywords:** ALC-0315, biodistribution, mRNA-LNP, pharmacokinetics, spike protein

## Abstract

**Aim:**

Lipid nanoparticle (LNP)-encapsulated mRNAs (mRNA-LNPs) are being explored for various prophylactic and therapeutic applications. However, the relationship between the pharmacokinetics (PK) of lipid, mRNA, and expressed protein across tissues remains poorly understood. This study aimed to perform a biodistribution study to quantitatively assess this relationship.

**Methods:**

Spike protein encoding mRNA was encapsulated in LNPs formulated with ALC-0315. mRNA-LNP was administered intravenously in mice. Biodistribution of ALC-0315, mRNA and expressed spike protein was determined in plasma and several tissues using LC–MS/MS, RT-qPCR and ELISA, respectively. Anti-spike protein IgM and IgG titers were also quantified using ELISA.

**Results:**

ALC-0315 lipid was cleared rapidly from plasma but persists in tissue for several weeks post dosing, with highest uptake seen in liver and spleen, and the lowest in muscle and brain. Unlike lipid, the highest mRNA exposure was observed in spleen followed by liver. Spike protein expression was detected within minutes of dosing and peaked at 6 h. Maximum protein expression was observed in liver, followed by spleen, heart, kidney and lung. A robust humoral immune response was triggered against spike protein, with IgM titers detected as early as 24 h and IgG titers detected on day 7.

**Conclusion:**

The PK of lipid, mRNA, and protein components differ significantly. The plasma PK for all three analytes differed significantly from tissue PK. Synthetic ionizable lipid ALC-0315 persisted in tissues for several weeks post dosing. After intravenous dosing, mRNA-LNP was found to be rapidly taken up by the tissues, and protein expression detected within one hour in all tissues.

**Supplementary Information:**

The online version contains supplementary material available at 10.1007/s11095-026-04086-4.

## Introduction

Following its discovery in the 19th century, RNA has long been recognized for its primary role in protein synthesis. However, its therapeutic potential has only recently been realized through the clinical success of mRNA containing lipid nanoparticle (LNP) vaccines against COVID-19, such as Cominarty (Pfizer-BioNTech) and Spikevax (Moderna) [1]. Lipid nanoparticles play a critical role in enabling the therapeutic viability of mRNA by shielding it from degradation and efficiently delivering it to target cells. Thus, the mRNA-LNP platform offers the ability to transiently express almost any protein *in-vivo* without genome integration. The favorable safety profile, high degree of customizability, accelerated development potential, and rapid, scalable manufacturing make mRNA-LNP a highly attractive modality. In fact, it is being actively explored for a wide range of prophylactic and therapeutic applications, including vaccines against infectious diseases, protein replacement therapies, personalized cancer vaccines, immunosuppressive therapies, antibody-based therapies, *in-vivo* gene editing and *in-vivo* CAR T therapies [2].

Despite the significant surge in research interest in recent years, most studies involving mRNA-LNP have focused on optimizing the formulations [3, 4] or assessing pharmacodynamic (PD) endpoints in preclinical species [5]. Very little effort has been directed towards understanding their pharmacokinetics (PK). A thorough understanding of the PK of mRNA LNPs and factors influencing it is essential for clinical success of this modality as it supports optimal formulation design, first in human dose selection, and safety as well as efficacy predictions. Recently, multiple studies have begun to address this gap by assessing the PK of mRNA LNPs following different routes of administration and dosing regimens [6, 7]. While these studies contribute valuable insights, most utilize reporter mRNAs to evaluate only transgene expression in a qualitative or semi-quantitative manner.

For mRNA LNP therapies the dose-exposure–response relationship can be more complex than that of traditional drugs. This complexity arises from the interplay of multiple processes involving the three components – the lipid nanoparticle, mRNA payload and expressed protein. The biodistribution and endosomal escape capability of lipid nanoparticles dictates mRNA distribution *in-vivo* and its delivery efficiency [8]. Once inside cells, mRNA half-life and translational efficiency are influenced by its structural elements such as untranslated regions (UTR), poly A tail, 5’ cap, modified nucleotides, and the gene of interest sequence. These mRNA properties, along with cell-intrinsic properties determine the magnitude and duration of protein expression [9]. Additionally, the signal peptide dictates whether the translated protein will be secreted, anchored to the membrane, or retained intracellularly. Once expressed, the protein follows its intrinsic PK behavior, which can result in significant variability in PK of various expressed proteins even when their mRNAs are delivered via the same LNP system. Therefore, to thoroughly understand the PK of mRNA-LNPs it is crucial to gain quantitative insights into the disposition of all its components (i.e., the LNP carrier, mRNA and translated protein) as well as their interplay. In this study, we address this critical knowledge gap by generating detailed quantitative PK data for the ionizable lipid, encapsulated mRNA, and expressed protein in blood and different tissues following mRNA-LNP administration in mice.

While a growing number of studies have begun to examine the PK of mRNA-LNP components individually, they often quantify biodistribution in limited number of tissues [4] or rely on labelling strategies [6]. Labelling strategies can provide misleading PK data as they might alter intrinsic PK of the molecule and often reflect the PK of the label itself and not the actual molecule at later timepoints. A recent study by Ci *et al.* [10] provides very valuable insights into the tissue biodistribution of Lipid 5 LNPs, encapsulated mRNA, and the expressed secretory protein Factor IX across multiple tissues in rats. Since LNPs with different lipid composition can have significantly different kinetics and altered mRNA distribution, it is important to quantitatively characterize the PK of mRNA LNPs made with various combination of lipids [11]. Such data is indispensable to gain a clear understanding of the contribution of individual components of mRNA-LNP towards their pharmacology.

In this study, we have quantitatively characterized the PK of mRNA-LNPs formulated with the ionizable lipid ALC-0315. Specifically, we measured the concentration of ionizable lipid, mRNA and expressed protein with LC–MS/MS, RT-qPCR and ELISA, respectively, post intravenous administration of mRNA-LNP. The LNPs were composed of the ionizable lipid ALC-0315, helper lipid distearoylphosphatidylcholine (DSPC), 1,2-dimyristoyl-rac-glycero-3-methoxypolyethylene glycol-2000 (DMG-PEG-2000) and cholesterol. mRNA expressing spike protein was used for our study as it is a membrane anchored protein, allowing for evaluation of tissue specific expression kinetics. Contrary to secreted proteins which rapidly diffuse into circulation, membrane bound proteins remain localized at site of expression allowing for more accurate understanding of transgene expression across tissues. The spike protein, while primarily membrane-bound, also undergoes limited shedding, resulting in detectable levels in plasma [12]. Since spike protein has been extensively studied, established assay tools are available to support quantification. We also studied the temporal profile of humoral immune response (anti-spike IgM and IgG) post IV dose of spike protein expressing mRNA LNPs. The PK data reported here provides novel insights into the systemic exposure and tissue accumulation of ALC-0315, mRNA distribution, and tissue specific protein translation, along with temporal profile of the humoral immune response to the expressed protein post intravenous dosing of mRNA-LNP.

## Methods

### mRNA Production and Characterization

The mRNA construct was designed to mimic the structural features of the Pfizer-BioNTech COVID-19 vaccine, ensuring efficient translation and robust protein expression. Specifically, the mRNA sequence included a 5′ untranslated region (UTR) derived from human α-globin RNA and a 3′ UTR composed of two regulatory elements from the amino-terminal enhancer of split (AES) mRNA and mitochondrial-encoded 12S ribosomal RNA. A 110-nucleotide segmented poly(A) tail was added to the 3′ end. To generate mRNA by *in vitro* transcription (IVT), the complete mRNA sequence was first cloned into a plasmid backbone. The plasmid was linearized using HindIII-HF (Cat# R3104, New England Biolabs) by incubating for 2 h at 37°C, providing a linear DNA template for IVT. Linearized DNA was purified by phenol–chloroform extraction to ensure removal of RNases and other impurities. mRNA was synthesized *in vitro* using the HIscribe™ T7 mRNA Kit (Cat# E2080, New England Biolabs) incorporating Cleancap Reagent AG and N1-methylpseudouridine (m1Ψ) (Cat# N1081, Tri Link Biotechnologies) to produce capped, tailed and chemically modified mRNA encoding the spike protein. The synthesized IVT RNA was run on a gel to confirm expected size of mRNA product and verify its integrity. Briefly, 5 µl of RNA sample (100 ng/µl) and 4 µl of RiboRuler High Range RNA Ladder (Cat#SM1821, Invitrogen) were mixed with 2 × RNA loading dye (#R0641, Invitrogen). Both RNA samples and ladder mixed with loading dye were incubated at 70°C for 10 min to denature mRNA followed by 3-min incubation on ice. The denatured samples and ladder were loaded onto an unstained 1% agarose gel and was run at 120 V for 40 min. Post electrophoresis, the gel was stained by incubating in 1 × SYBR Gold staining solution (Invitrogen #Cat no. S11494) on a gel shaker for 20 min at room temperature, protected from light. The stained gel was visualized in the Bio-Rad Chemidoc Imaging system (#3373246, Bio-Rad) under SYBR gold settings. The produced mRNA was stored in −80°C until needed.

### Lipid Nanoparticle Production

**Fig. 1:**
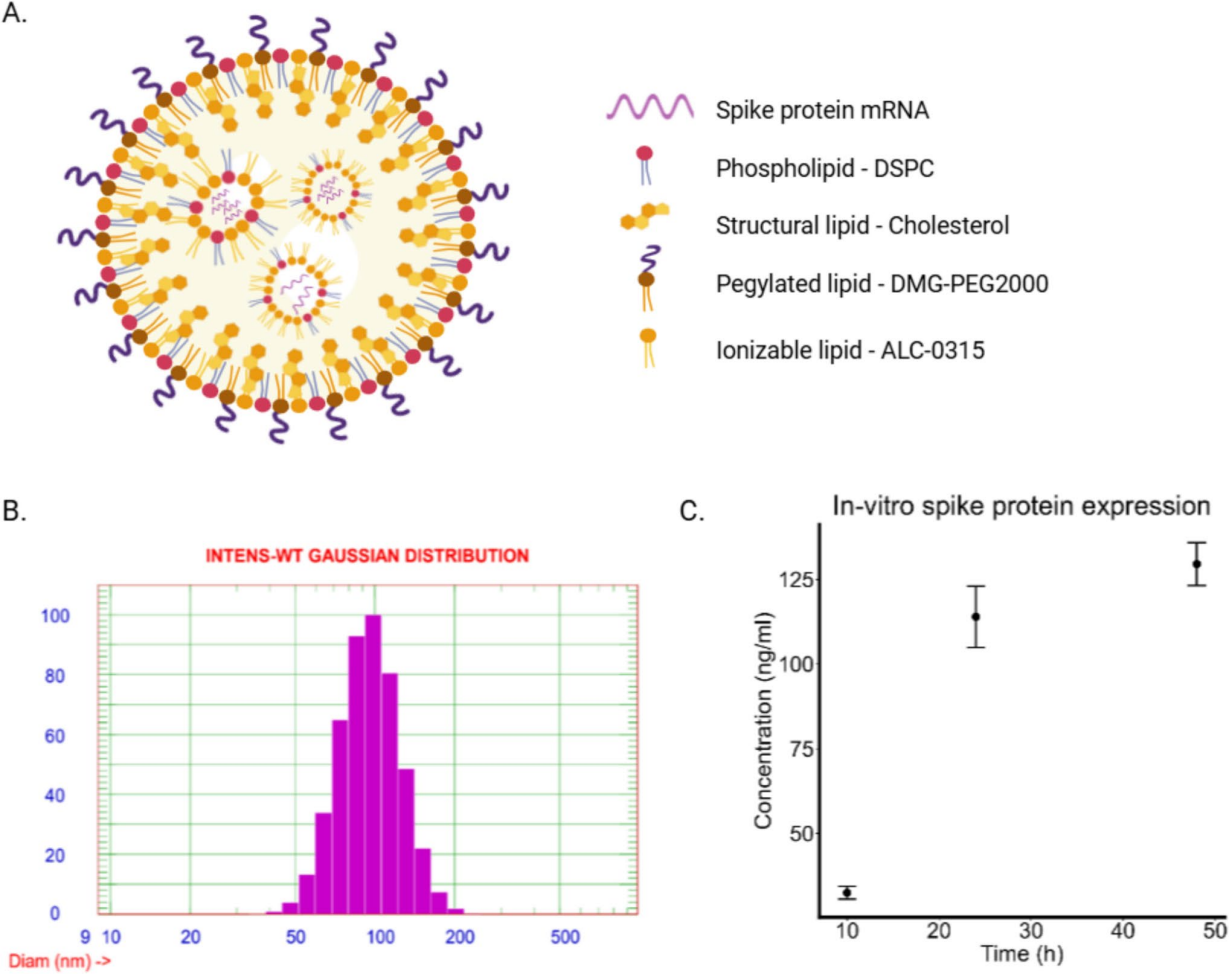
(**A**) Schematic illustration of the LNP structure. The ionizable lipid (ALC-0315) becomes positively charged in acidic pH during LNP formation and forms stable complexes with the negatively charged spike protein mRNA in the core of the LNPs. The phospholipids, cholesterol and PEG-lipids form a stable shell outside and help maintain LNP integrity and stability. (**B**) DLS analysis of produced mRNA-LNP revealed a mean diameter of 98 nm and a homogenous formulation with PDI 0.07. (**C**) *In vitro* protein expression kinetics in HEK293T cells following transfection with 500 ng mRNA LNP. Increasing concentrations of spike protein with time were detected in cell supernatants with ELISA confirming efficient expression of full length properly folded protein.

The synthesized IVT mRNA was encapsulated within a lipid nanoparticle by microfluidic mixing. Briefly, a lipid mix was prepared by dissolving ALC-0315, distearoylphosphatidylcholine (DSPC), cholesterol and 1,2-dimyristoyl-rac-glycero-3-methoxypolyethylene glycol-2000 (DMG-PEG-2000) in ethanol at a molar ratio of 50:10:38.5:1.5 (Fig. 1A). The IVT synthesized mRNA was dissolved in 50 mM citrate buffer (pH 4). The lipid and mRNA concentrations were calculated based on an N:P ratio of 6:1. The lipid mix, and aqueous solution of mRNA were rapidly mixed at a volumetric flow rate ratio of 3:1 and total flow rate of 5 ml/min in a microfluidic mixer (Flex M, Precigenome) allowing for encapsulation and self-assembly of lipid nanoparticles. The produced lipid nanoparticles were immediately dialyzed against PBS (pH 7.4) in Slide-A-Lyzer cassettes (10 K MWCO) for 12–15 h to remove ethanol and exchange buffer. The dialyzed LNPs were then concentrated to desired mRNA concentration using Amicon Ultra-15 centrifugal filter units 100 kDa (Cat #UFC910024). The concentrated LNPs were sterile filtered through a 0.22 µm filter and stored at 4°C for future use.

### Lipid Nanoparticle Characterization

The particle size distribution of the lipid nanoparticles was determined by dynamic light scattering (DLS) using a submicron particle sizer Nicomp 380 (Particle size systems). LNP samples were diluted in nuclease free PBS and loaded onto the machine in disposable cuvettes. Measurements were conducted at 23°C and mean diameter and PDI (poly dispersity index) of produced LNPs were obtained. The Quant-iT™ Ribogreen™ RNA Assay Kit (Cat #R11490, Invitrogen) was used with a modified protocol to measure mRNA concentration of synthesized LNPs as well as their encapsulation efficiency. Briefly, mRNA standards ranging from 2.5 to 0.1 µg/ml were prepared in Triton buffer (2% Triton X-100 in TE buffer). LNP samples were diluted in PBS to ensure concentrations were within the linear range of the standard curve. A working solution of Ribogreen reagent was prepared by diluting stock reagent 100 × in TE buffer. LNP samples were diluted in plain TE buffer to measure free RNA, while Triton X-100 was added to LNPs separately to lyse nanoparticles and measure total RNA concentrations. mRNA standards, blanks, and LNP samples were incubated with equal volume of Ribogreen reagent in black, flat bottom 96 well assay plates (#Cat3916, Corning) and fluorescence was measured with FilterMax F5 multimode microplate reader set to excitation wavelength of 485 nm and emission wavelength of 535 nm. The encapsulation efficiency was calculated using the following equation.

$$Encapsulation efficiency (\%)= \frac{Total mRNA-Free mRNA}{Total mRNA}*100$$

### Evaluation of *In-Vitro* Protein Expression

HEK293T cells (ATCC CRL 3216) were transfected with mRNA-LNP to confirm spike protein expression *in vitro* prior to proceeding with animal studies. 1E5 HEK293T cells were seeded on 24 well plates and incubated for about 20–24 h at 37°C in 5% CO₂. Once cells reached about 80% confluency, mRNA LNPs corresponding to a 500 ng mRNA dose were added directly to each well. 30 µl of culture media was collected at 10, 24 and 48 h post transfection and replaced with fresh media. Secreted spike protein in media was measured using ELISA, as described below.

### Biodistribution Study in Animals

**Fig. 2:**
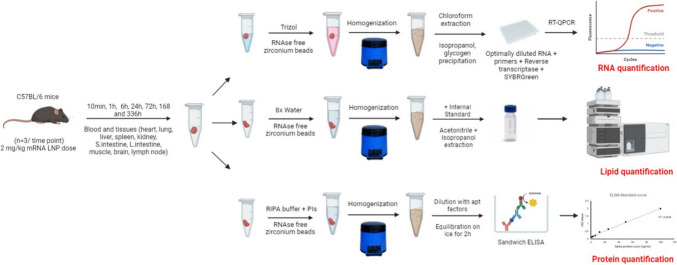
Schematic illustration of the whole-body biodistribution study in mice. Mice were grouped (*n* = 3 per time point) and administered mRNA-LNP at mRNA dose of 2 mg/kg. At designated time points, blood and major tissues were collected and subdivided into three parts. Each fraction was processed separately for the quantification of mRNA, the ionizable lipid ALC-0315, and the expressed protein, as indicated in the figure.

The animal protocol was approved by the Institutional Animal Care and Use Committee of the University at Buffalo. The invivo study design is shown in Fig. 2. Briefly, 5–6-week-old male C57BL/6 J mice (Cat#000664, Jackson Laboratories) weighing 20–25 g were dosed with 2 mg/kg spike protein expressing mRNA LNP intravenously via the penile vein. At 10 min, 1 h, 6 h, 24 h, 72 h, 168 h and 336 h, blood was collected via portal vein and mice were euthanized by exsanguination (n = 3 per time point). 200 µl of blood was added to TRI reagent BD (#Cat T3809, Sigma Aldrich) for mRNA quantification and stored at −80°C while rest of the blood was centrifuged at 2000rcf for 20 min to obtain plasma. Plasma samples were stored at −20°C for lipid and protein quantification. Tissues including heart, lung, liver, spleen, kidney, small intestine, large intestine, muscle, brain and lymph node were collected, blot dried and split into 3 parts: (1) ~ 20 mg was added to RNAlater solution (#Cat AM7020, Invitrogen) and stored at 4°C for IVT RNA quantification, (2) ~ 20 mg was snap frozen in liquid nitrogen and stored at −80°C for ionizable lipid quantification, (3) rest of the tissue was snap frozen in liquid nitrogen and stored at −80°C for expressed spike protein quantification.

### Lipid Quantification by LC/MS/MS

To prepare samples for LC/MS/MS analysis, frozen tissue samples were weighed (~ 20–50 mg) and diluted with 8 × volume of water. The tissues were then homogenized in 5 ml tubes with 3–4 3 mm zirconium beads in the bullet blender homogenizer (Next Advance) at a speed of 12 for 5 min or until tissues were homogenized completely. 50 µl of sample homogenate was spiked with 1 ng/ml of internal standard (DLin-DMC3) and extracted with 400 µl of acetonitrile/isopropanol (v/v 50:50) to precipitate proteins. The samples were centrifuged at 4000 rpm for 10 min and the supernatant was transferred to LCMS vials for analysis using the method described below.

The ionizable lipid ALC-0315 was quantified by liquid chromatography with tandem mass spectrometry detection (LC/MS/MS). The quantification method for ALC-0315 was developed on a Qtrap 6500 + system (AB Sciex LLC) with a XSelect CSH C18 column 3.5 µm (Waters). First, a Q1 precursor ion scan was performed across 500 to 900 m/z values. The precursor ion for ALC-0315 was identified at 766.6 m/z. Next, a product ion scan was conducted at a collision energy of 70 eV to identify suitable fragment ions for quantification. Product ions were observed at m/z 748.6, 510.5, 438.2. The product ion at m/z 748.6 was finalized for use in quantification method based on its superior sensitivity and reproducibility. The LC separation was conducted at a flow rate of 0.2 ml/min over a 16 min runtime at 70°C column temperature. ALC-0315 was quantified in positive ion mode using multiple reaction monitoring (MRM) with the m/z transition 766.6 → 748.6.

### mRNA Quantification by RT-qPCR

About 200 µl of blood was immediately added to 750 µl of TRI Reagent BD with 20 µl 5 N acetic acid. The samples were kept at RT for 5 min followed by storage in −80°C until analysis. About 20 mg of tissues were collected in RNAlater solution and stored at 4°C until analysis. Total RNA was extracted from collected blood and tissue samples with TRI reagent BD (#Cat T3809, Sigma Aldrich) and TRIzol reagent (#Cat 15,596,026, Invitrogen) respectively according to manufacturer’s instructions. The precipitated RNA was treated with DNase I and resuspended in nuclease free water and stored in −80°C until further analysis.

IVT mRNA from extracted total RNA was quantified using a 1 step RT-qPCR method developed in-house with the SuperScript III Platinum SYBR Green One-Step qRT-PCR Kit (#Cat 11,736,059, Invitrogen). Multiple primer pairs were designed and screened to identify primers which provided optimal amplification. A primer pair resulting in a slope of −3.1 and corresponding amplification efficiency of 109% was chosen. Selected primer pairs were further validated using total RNA extracted from control tissues to ensure absence of nonspecific amplification. mRNA standard curves and QCs were prepared in nuclease free water for each tissue. Blank tissue matrix were spiked with known quantity of LNP and extraction efficiency (EE) was calculated for each tissue using the following formula.

$$EE (\%)= \frac{Amount of mRNA quantified}{Amount of mRNA spiked}*100$$

Standards, QCs, samples, mouse RNA (negative control) and TE buffer (blank) were added to 96 well PCR plates (#Cat HSL9605, Biorad) and were run on Biorad CFX96 thermocycler. The results were analyzed on CFX Maestro software (Biorad). Measured mRNA values were normalized for extraction efficiency.

### Spike Protein Quantification by ELISA

For spike protein quantification in tissues, frozen samples were weighed and homogenized in 5 × RIPA buffer supplemented with protease and phosphatase inhibitors. Tissue homogenization was performed using 4–5 zirconium beads (3 mm diameter) in a Bullet Blender homogenizer (Next Advance) at speed 12 for 5–10 min or until tissue was completely homogenized. Standard curves and QCs were prepared in the same tissue matrix as samples. Homogenized tissue samples along with standards, QCs and blanks were incubated in 4°C overnight. The next day, tissue homogenates were centrifuged at 15000 g for 15 min at 4°C and supernatant was used for protein quantification. Plasma and tissue samples were diluted in corresponding blank plasma or tissue homogenate to ensure that sample concentrations were within linear range of the standard curve. The SARS-CoV-2 Spike Trimer (Wild Type) Specific ELISA Kit (#Cat RAS-A115, Acro biosystems) was used for quantification of spike protein according to manufacturer’s instructions.

## Data Analysis

Non-Compartmental analysis of the collected data was conducted in R using PKNCA package. Given the sparse terminal sampling design, Bailer’s method was used to calculate AUC and associated SE.

### Anti-Spike Protein IgM and IgG titer Measurement

A protocol similar to the one previously used in by lab to quantify anti-AAV humoral response was employed [13]. Briefly, spike protein trimer was dissolved in coating buffer and 384 well ELISA plates were coated with spike protein at 4°C overnight. The next morning, the wells were blocked with a blocking buffer for 2 h at room temperature. Post blocking, 30 µl plasma samples from various time points were added at dilutions ranging from 10 × to 100,000 × in triplicates. The plate was then incubated with samples for 2 h on a plate shaker at room temperature. Post incubation with samples, Fab2 goat anti-mouse IgG Fc (#Cat A90-239AP)and Fab2 goat anti-mouse IgM (#CatA90-140APAP) conjugated secondary antibodies (1000 × dilution in wash buffer) were used to detect anti-spike protein IgG and IgM antibodies, respectively. Post 60 min incubation with secondary antibodies, the substrate PNPP (p-nitrophenyl phosphate) solution was added, and the absorbance values were read in plate reader at 405 nm. The highest dilution with a detectable signal was considered as the titer at that time point. The plates were washed thrice with wash buffer (PBS with 0.05% Tween 20) and thrice with water between each step.

## Results

### Characterization and *In Vitro* Functional Validation of mRNA-LNP

Capped and tailed mRNA encoding the SARS-CoV-2 spike protein was successfully synthesized using *in vitro* transcription. The transcribed mRNA appeared as a distinct, sharp band at approximately 4000 bp on RNA gel, with no visible smearing, confirming the production of full-length, non-degraded RNA. The synthesized mRNA was then encapsulated into lipid nanoparticles (LNPs) using microfluidic mixing with the Flex-M instrument. The produced mRNA LNPs had an average diameter of 98.4 nm with a PDI of 0.07 (< 0.2) (Fig. 1B). The encapsulation efficiency of the produced LNPs, as determined by Ribogreen assay, was 89%. Produced mRNA LNPs were then transfected into HEK293T cells to verify expression of intact spike protein. Presence of expressed spike protein in cell culture supernatant at 10, 24 and 48 h post transfection was confirmed with ELISA. Spike protein levels increased steadily over time and continued to rise to the final time point at 48 h (Fig. 1C). Since spike protein is not a secretory protein, its presence in the culture supernatant is likely attributable to shedding from the plasma membrane of transfected cells [12]. About 125 ng/ml of spike protein was detected in the supernatant 48 h post transfection with 250 ng mRNA LNPs, confirming successful transfection of cells and translation of protein within cells.

### Biodistribution of ALC-0315 Lipid and Encapsulated mRNA in Mice Following Intravenous Administration of mRNA-LNP

The LCMS method to quantify ALC-0315 was established successfully. Standard curves were initially prepared in each tissue matrix and compared to assess matrix effects. The overlap of calibration curves across all matrices indicated minimal matrix interference. Therefore, the final standard curve was generated in control plasma, while quality control (QC) samples were prepared in their respective tissue matrices. Sample concentrations were accepted for analysis only when corresponding QC samples met predefined acceptance criteria (± 15%). Representative standard curve to measure ALC-0315 in plasma and tissues is provided in Supplementary Fig. 1. Intra- and inter-day precision and accuracy for all calibration standards and quality control (QC) samples in plasma and all tissue matrices were within ± 15% of their nominal concentrations. The quantitation range was 1–2000 ng/mL, with QC concentrations set at 800, 80, 8, and 2 ng/ml.

**Fig. 3:**
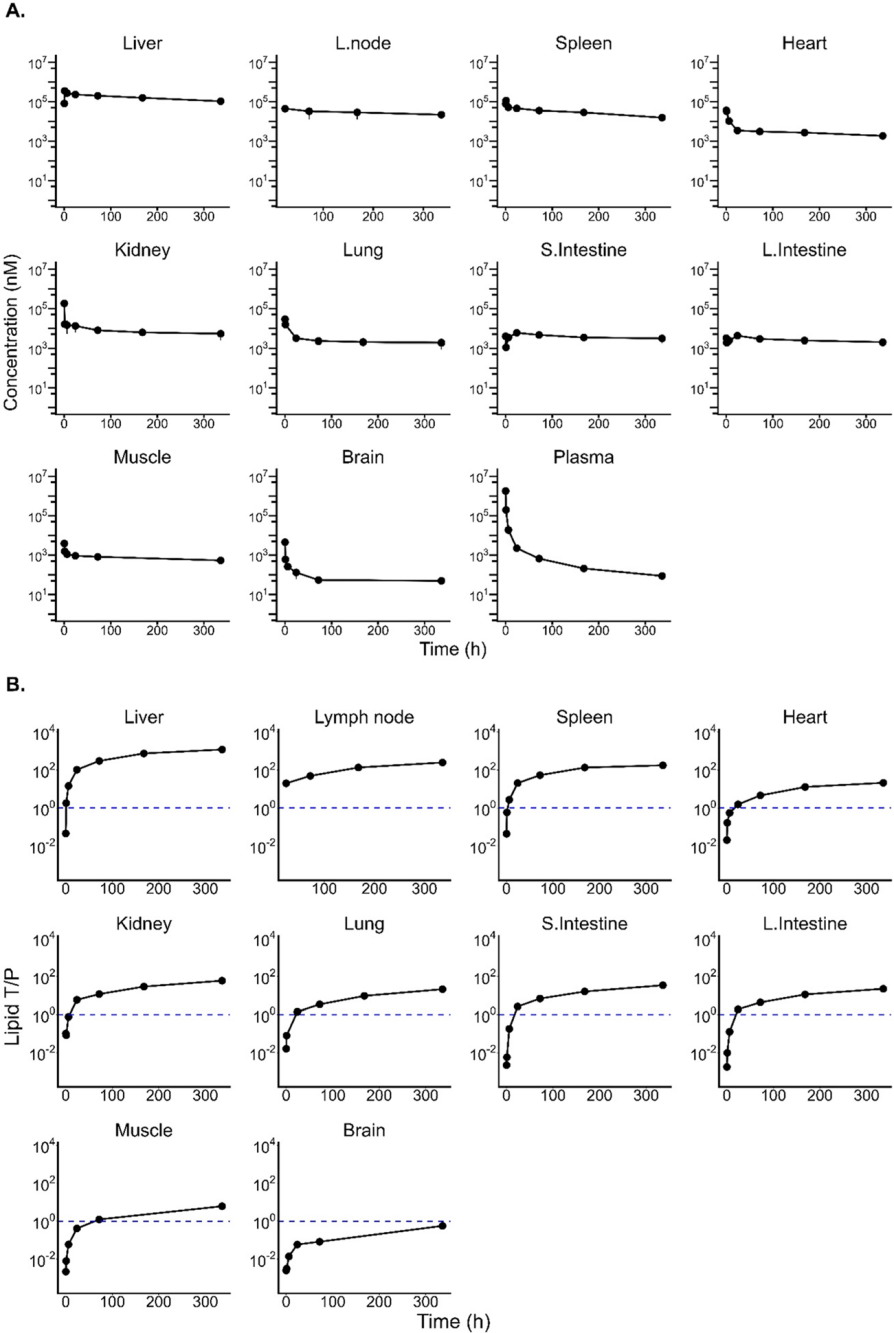
Whole body PK of ALC-0315. (**A**) Biodistribution of ALC-0315 in major tissues and plasma following intravenous dosing of 2 mg/kg mRNA LNP in C57BL/6 mice. Solid circles represent mean concentration and error bars indicate geometric SD. MW of ALC-0315 is 766.2 mol/g. (**B**) Mean tissue/plasma (T/P) concentration ratios of ALC-0315. Horizontal dashed line represents ratio of 1. Significant accumulation was observed in all tissues with T/P ratios crossing 1 in all tissues except brain.

Following intravenous administration of LNP, ALC-0315 was quickly cleared from plasma, characterized by a steep decline in plasma concentrations during alpha phase (Fig. 3A). Lipid exhibited rapid and widespread distribution across all examined tissues, reaching peak concentrations (${T}_{max}$) as early as 10 min (the earliest time point assessed) across most organs (Fig. 3A). Notably, only liver, spleen and intestine (Fig. 3A) demonstrated delayed ${T}_{max}$ values, with peak levels detected at 1-h post-dose. These findings suggest that LNPs are rapidly taken up by all tissues within minutes of their administration. The kinetics of ALC-0315 differed markedly between plasma and tissues (Figs. 3A and 6A), highlighting that reliance on plasma data alone may lead to misrepresentation of actual tissue exposure. While plasma exhibited highest concentration among all tissues at 10 min post-dose, plasma concentration declined rapidly thereafter, with the liver exhibiting the highest ALC-0315 concentrations at all subsequent time points. Substantial levels of ALC-0315 were still detected in tissues two weeks after dosing, indicating prolonged retention and slow clearance from tissue compartments.

The highest tissue exposure of ALC-0315, as measured by AUC₀₋ₜ, was observed in the liver (5.76 × 10⁷ nM·h), spleen (9.98 × 10⁶ nM·h), and kidney (2.55 × 10⁶ nM·h), corresponding to tissue-to-plasma AUC ratios of 37.09, 6.43 and 1.64, respectively (Table I). Although ALC-0315 concentrations in lymph node were not available for early time points, concentrations measured post 24 h were comparable to those in spleen, suggesting substantial distribution to lymphatic tissue. In contrast, the lowest exposures post systemic administration were observed in muscle(2.48 × 10^5^ nM·h) and brain (2.57 × 10^4^ nM·h) with tissue-to-plasma AUC ratios of 0.16 and 0.02 respectively (Table I). Almost 2,000-fold difference in ALC-0315 exposure between liver and brain highlight the strong tropism of LNPs to liver as well as restricted access to brain tissue post intravenous administration. The tissue-to-plasma ratio *vs.* time profiles for ALC-0315 (Fig. 3B) demonstrate a steep rise within the first 24 h, reflecting rapid tissue uptake coupled with a sharp decline in plasma concentrations. This was followed by a more gradual increase in the ratio over time, indicative of sustained tissue retention relative to continued plasma clearance. Notably, ALC-0315 concentrations in the brain remained consistently lower than plasma levels throughout the study period, indicating limited penetration of LNPs across the blood–brain barrier.

**Table I:** PK Parameters for ALC-0315 Lipid Obtained using Noncompartmental Analysis (NCA) Following Intravenous Administration of 2 mg/kg mRNA-Loaded Lipid Nanoparticles (LNPs) in C57BL/6 Mice

	${C}_{max}$(SE) (nM)	${T}_{max}$(h)	${T}_{1/2}$(h)	${\lambda }_{z}$(1/h)	${AUC}_{0-t}$(**SE)** (nM·h)	${~}^{T}\!\left/ \!{~}_{P}\right.$ ratio
Plasma	1.80 × 10⁶ (5.02 × 10^5^)	0.167	93.8	0.00739	1.55 × 10⁶ (2.82 × 10^5^)	-
Heart	3.66 × 10^4^ (1.64 × 10^2^)	0.167	356	0.00195	1.06 × 10⁶ (4.47 × 10^4^)	0.680
Lung	2.90 × 10^4^ (1.07 × 10^3^)	0.167	1.03*10^3	0.000670	8.65 × 10^5^ (7.3 × 10^4^)	0.560
Liver	3.54 × 10^5^ (1.61 × 10^4^)	1.00	348	0.00199	5.76 × 10⁷ (2.15 × 10^6^)	37.1
Spleen		1.00	207	0.00335	9.98 × 10⁶ (2.95 × 10^5^)	6.43
	1.15 × 10^5^ (9.95 × 10^3^)					
Kidney	1.85 × 10^5^ (1.44 × 10^4^)	0.167	482	0.00144	2.55 × 10⁶ (2.02 × 10^5^)	1.64
Small Intestine	5.94 × 10^3^ (7.62 × 10^2^)	24.0	481	0.00144	1.28 × 10⁶ (1.04 × 10^5^)	0.830
Large Intestine		24.0	505	0.00137	8.67 × 10^5^ (4.63 × 10^4^)	0.560
	4.25 × 10^3^ (7.02 × 10^2^)					
Muscle	3.92 × 10^3^ (5.20 × 10^1^)	0.167	414	0.00168	2.48 × 10^5^ (2.09 × 10^4^)	0.160
Brain	4.51 × 10^3^ (5.99 × 10^1^)	0.167	134	0.00517	2.57 × 10^4^ (2.06 × 10^3)	0.0200
Lymph node		24.0	452	0.00153	-	-
	4.48 × 10^4^					

Data are presented for plasma and 10 tissues. Data was not available for earlier time points in lymph node and hence ${AUC}_{0-t}$ ratios and tissue-to-plasma (T/P) ratio could not be calculated. Also, true ${C}_{max}$ and ${T}_{max}$ for lymph nodes might be different as first collected time point for lymph node was 24 h. ${C}_{max}$ refers to maximum observed concentration, ${T}_{max}$ refers to timepoint with maximum observed concentration, ${T}_{1/2}$ refers to elimination half-life, ${\lambda }_{z}$ is terminal slope, ${AUC}_{0-t}$ is area under the concentration time curve from time 0 to last quantified concentration, and T/P ratio refers to ratio of ${AUC}_{0-t}$ value in tissue and plasma. MW of ALC-0315 used for calculations was 766.2 g/mol. All values have been rounded off to 3 significant figures

**Fig. 4:**
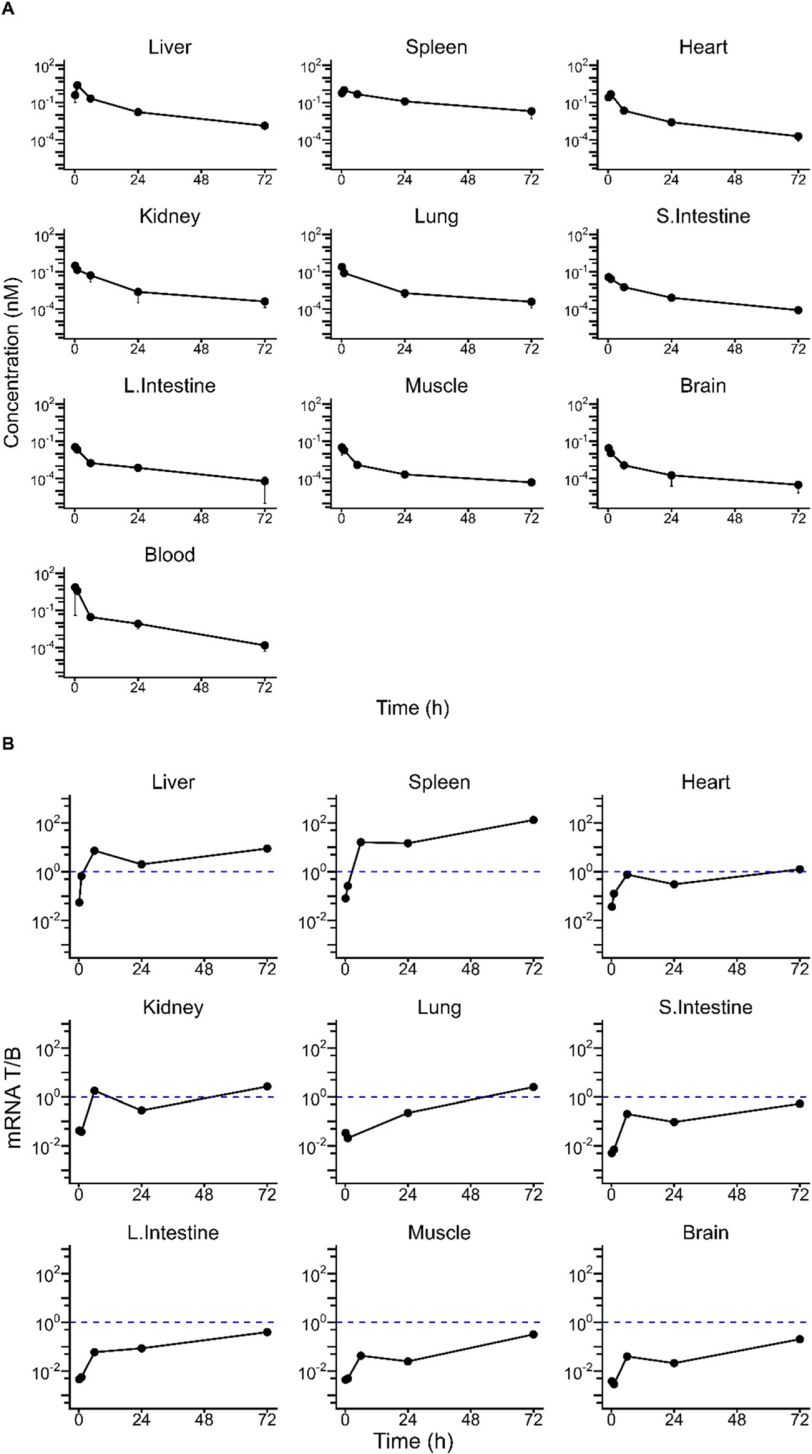
Whole body PK of mRNA. (**A**) PK profiles of mRNA in major tissues and plasma following intravenous dosing of 2 mg/kg mRNA-LNP in C57BL/6 mice. Solid circles represent mean concentration and error bars indicate geometric SD. MW of mRNA is 1,454,860 g/mol. (**B**) Mean tissue/blood (T/B) concentration ratios of mRNA. Horizontal dashed line represents T/B ratio of 1. Maximum mRNA localization was seen in spleen and liver.

To quantify mRNA, an RT-qPCR method was developed. Multiple primer pairs were screened, and the optimized primer set produced a standard curve with a slope of –3.11, indicating acceptable DNA amplification efficiency. A representative standard curve has been provided in Supplementary Fig. 2. Following mRNA-LNP administration, rapid uptake of mRNA was observed in tissues within the first few minutes post-administration, similar to the early distribution pattern of ALC-0315, confirming rapid delivery of mRNA-LNPs to tissues shortly after intravenous dosing (Fig. 4A). ${T}_{max}$ of mRNA in most tissues was found to be 10 min while liver, spleen and heart exhibited delayed peak levels reaching ${T}_{max}$ at 1 h. Unlike ALC-0315 which showed prolonged accumulation within tissues, mRNA was quickly cleared from tissues and was no longer detectable beyond 72 h. Highest mRNA exposure $({AUC}_{0-72h})$ was seen in blood (10.01 nM·h), spleen (11.71 nM·h) and liver (7.630 nM·h) with tissue-to-blood AUC ratios 1.17 and 0.76, respectively (Figs. 4B, 6B and Table II). Minimal mRNA distribution was observed in the intestine, muscle, and brain, as reflected by tissue-to-blood AUC ratios < 0.05 (Table II). Tissue-to-blood mRNA concentration ratio *vs.* time profiles (Fig. 4B) revealed that mRNA levels in tissues such as the small intestine, large intestine, muscle, and brain remained consistently lower than those in blood across all time points. In contrast, only the liver and spleen exhibited higher mRNA concentrations than blood beginning at 6 h post-dose, suggesting greater uptake in these organs. Distinct PK profiles of mRNA were observed in blood and tissues (Fig. 6B), underscoring the importance of independently evaluating mRNA kinetics in target tissues. Furthermore, the marked differences in the PK of the ionizable lipid (ALC-0315) and the encapsulated mRNA following mRNA-LNP administration highlight the need to assess both components separately to fully comprehend the biodistribution of mRNA-LNP.

**Table II:** PK Parameters for Spike Protein Encoding mRNA Following Intravenous Administration of 2 mg/kg mRNA-Loaded LNP in C57BL/6 Mice

	${C}_{max}$(SE) (nM)	${T}_{max}$(h)	${T}_{1/2}$(h)	${\lambda }_{z}$(1/h)	${AUC}_{0-t}$(SE) (nM·h)	${~}^{T}\!\left/ \!{~}_{B}\right.$ ratio
Blood	7.33 (4.21)	0.167	8.60	0.08	10.0(3.05)	
Heart	0.478 (0.042)	1.00	10.2	0.07	1.28 (0.136)	0.120
Lung	0.247 (0.078)	0.167	10.1	0.07	0.670 (0.197)	0.0700
Liver	2.52 (1.10)	1.00	9.70	0.07	7.63 (3.28)	0.760
Spleen	0.999 (0.169)	1.00	15.2	0.05	11.7 (1.52)	1.17
Kidney	0.309 (0.114)	0.167	8.90	0.08	1.00 (0.269)	0.100
Small Intestine	0.0370 (0.005)	0.167	11.3	0.06	0.160 (0.020)	0.0200
Large Intestine	0.0340 (0.007)	0.167	12.0	0.06	0.100 (0.016)	0.0100
Muscle	0.0320 (0.014)	0.167	15.3	0.05	0.070 (0.016)	0.0100
Brain	0.0280 (0.005)	0.167	13.6	0.05	0.050 (0.006)	0.0100

${C}_{max}$ refers to maximum observed concentration, ${T}_{max}$ refers to timepoint with maximum observed concentration, ${T}_{1/2}$ refers to elimination half-life, ${\lambda }_{z}$ is terminal slope, ${AUC}_{0-t}$ is area under the concentration time curve from time 0 to last quantified concentration, and T/P ratio refers to ratio of ${AUC}_{0-t}$ value in tissue and plasma. Spike protein encoding mRNA was 4279 bp long with MW of 1,454,860 g/mol. All values have been rounded off to 3 significant figures

### Biodistribution of Protein Expressed Following mRNA-LNP Administration

**Fig. 5:**
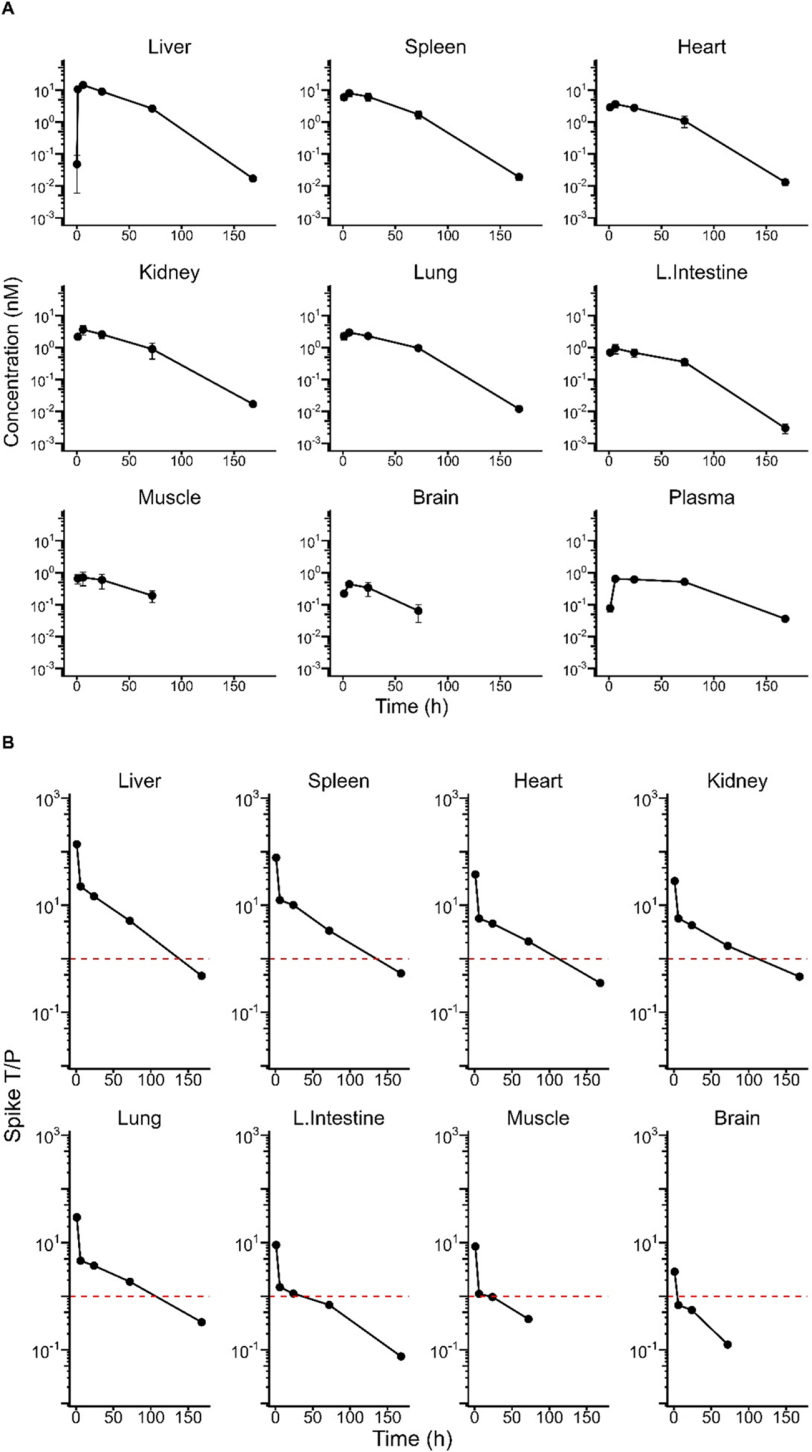
Whole body distribution of translated spike protein. (**A**) Biodistribution of spike protein in major tissues and plasma following intravenous dosing of 2 mg/kg mRNA-LNP in C57BL/6 mice. Solid circles represent mean concentration and error bars indicate geometric SD. MW of spike protein trimer is 414,000 g/mol. (**B**) Mean tissue/plasma (T/P) concentration ratios of spike protein. Horizontal dashed line represents T/P ratio of 1. Values greater than 1 at early timepoints indicates production and localization of spike protein within tissues. At later time points, T/P concentration ratios fall below 1 as protein is cleared from tissues and also undergoes shedding into plasma.

Spike protein expression was rapidly detected across all examined tissues, with quantifiable levels observed within 1 h post-dosing in all tissues (Fig. 5A). Notably, in the liver, spike protein was detectable in two animals as early as 10 min, indicating rapid onset of translation in liver. Spike protein concentrations increased across all tissues during the first 6 h post-dosing, with peak levels observed at 6 h in each tissue. Following this peak, a gradual decline in tissue concentrations was noted up to 72 h, after which a more rapid elimination phase was observed. At 336 h, spike protein was no longer detectable in all tissues, with only 2 of 3 animals showing measurable levels at 168 h timepoint. For data analysis, spike protein concentrations below the limit of quantification (BLQ) were replaced with LOQ/2 values for the corresponding tissues, in line with commonly accepted methods for managing censored PK data [14]. In muscle and brain, spike protein concentrations were not detectable at 168 h.

**Fig. 6:**
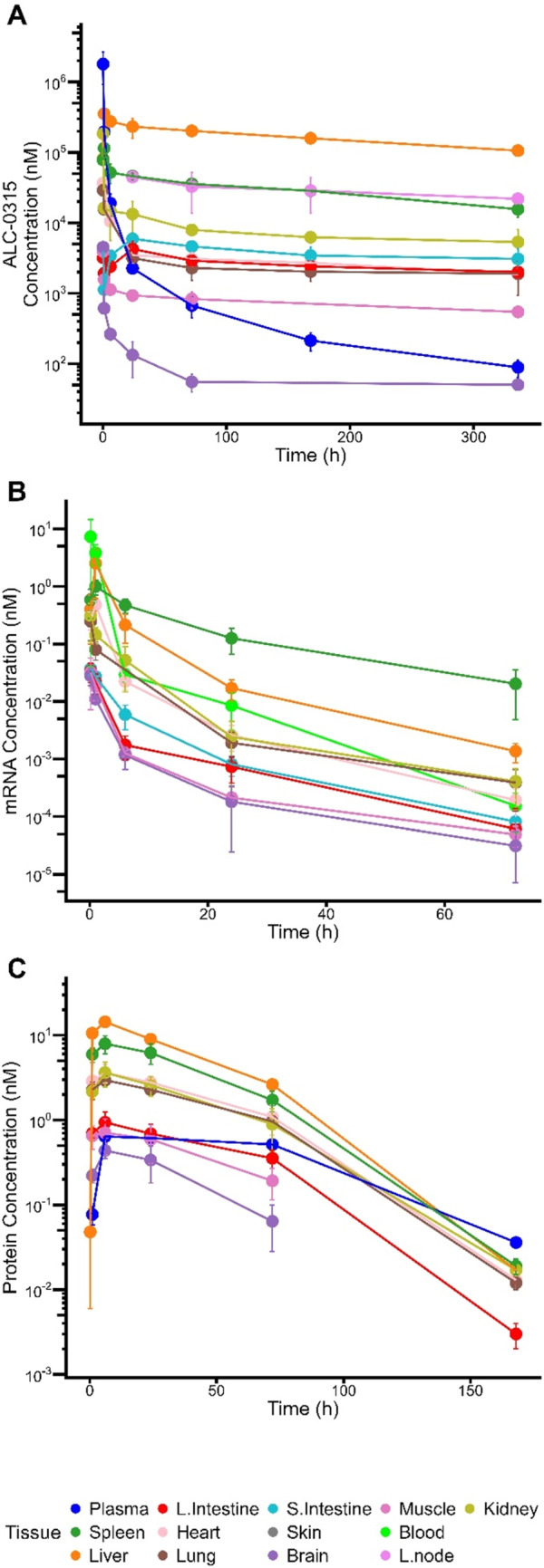
Pharmacokinetic profiles of (**A**) ALC-0315 lipid, (**B**) mRNA, and pharmacodynamic profile of (**C**) expressed spike protein across plasma/blood and major tissues. Profiles are overlaid to compare biodistribution and expression kinetics. ALC-0315 LNPs exhibited predominant accumulation in the liver, indicating strong hepatic tropism. mRNA showed the slowest clearance in the spleen. Correspondingly, spike protein expression peaked in the liver, followed by the spleen. Minimal LNP uptake and mRNA presence were observed in the brain, resulting in the lowest spike protein expression in this tissue.

As spike protein is membrane-bound, the expressed protein is expected to remain localized within tissues rather than being actively secreted into circulation, unless shed from the cell surface into plasma. Consistent with this, low levels of spike protein were detected in plasma at 1 h, followed by a marked increase at 6 h. Plasma concentration remained relatively stable until 72 h before declining. The slower elimination of spike protein from plasma compared to tissues may be attributed to ongoing shedding of membrane-bound protein into circulation at later time points (Figs. 5A, B and 6C). The liver exhibited the highest cumulative expression, with an AUEC of 574.13 nM·h and a tissue-to-plasma ratio of 10. This was followed by the spleen (368.2 nM·h), heart (184.5 nM·h), kidney (168.6 nM·h) and lung (155.1 nM·h), with tissue-to-plasma AUC ratios of 6.4, 3.2, 2.9 and 2.7, respectively. Spike protein expression in large intestine, muscle and brain was minimal, with tissue-to-plasma AUEC ratios below 1 (0.87, 0.71 and 0.29, respectively), suggesting little protein expression in these tissues. (Fig. 5B, Table III).

**Table III:** PK Parameters for Translated Spike Protein Following Intravenous Administration of 2 mg/kg mRNA-Loaded LNP in C57BL/6 Mice

	${C}_{max}$(SE) (nM)	${T}_{max}$(h)	${T}_{1/2}$(h)	${\lambda }_{z}$(1/h)	${AUEC}_{0-t}$(SE) (nM·h)	${~}^{T}\!\left/ \!{~}_{P}\right.$ ratio
Plasma	0.640 (0.0398)	6	33.4	0.021	57.40 (2.25)	-
Heart	3.61 (0.506)	6	17.9	0.038	184.5 (19.3)	3.21
Lung	2.95 (0.210)	6	18.3	0.038	155.1 (3.09)	2.70
Liver	14.4 (0.447)	6	15.5	0.045	574.8 (20.1)	10.0
Spleen	7.93 (1.08)	6	16.9	0.041	368.2 (38.9)	6.41
Kidney	3.62 (0.669)	6	19.3	0.036	168.6 (24.2)	2.94
Large Intestine	0.940 (0.178)	6	17.3	0.040	50.1 (5.54)	0.870
Muscle	0.710 (0.185)	6	-	-	32.4* (5.90)	0.710
Brain	0.440 (0.050)	6	-	-	16.6* (3.09)	0.290

${C}_{max}$ refers to maximum observed effect, ${T}_{max}$ refers to timepoint with maximum observed effect, ${T}_{1/2}$ refers to elimination half-life, ${\lambda }_{z}$ is terminal slope, ${AUEC}_{0-t}$ is area under the effect *versus* time curve from time 0 to last quantified effect, and T/P ratio refers to ratio of ${AUEC}_{0-t}$ value in tissue and plasma. Molecular weight of spike protein trimer is 414,000 g/mol. All values have been rounded off to 3 significant figures

^*^AUCs reported for muscle and brain are ${AUC}_{0-72h}$ since spike protein could not be detected in both tissues at 168 h, while those reported for other tissues is ${AUC}_{0-168h}$

### Humoral Immune Response to Spike Protein

**Fig. 7:**
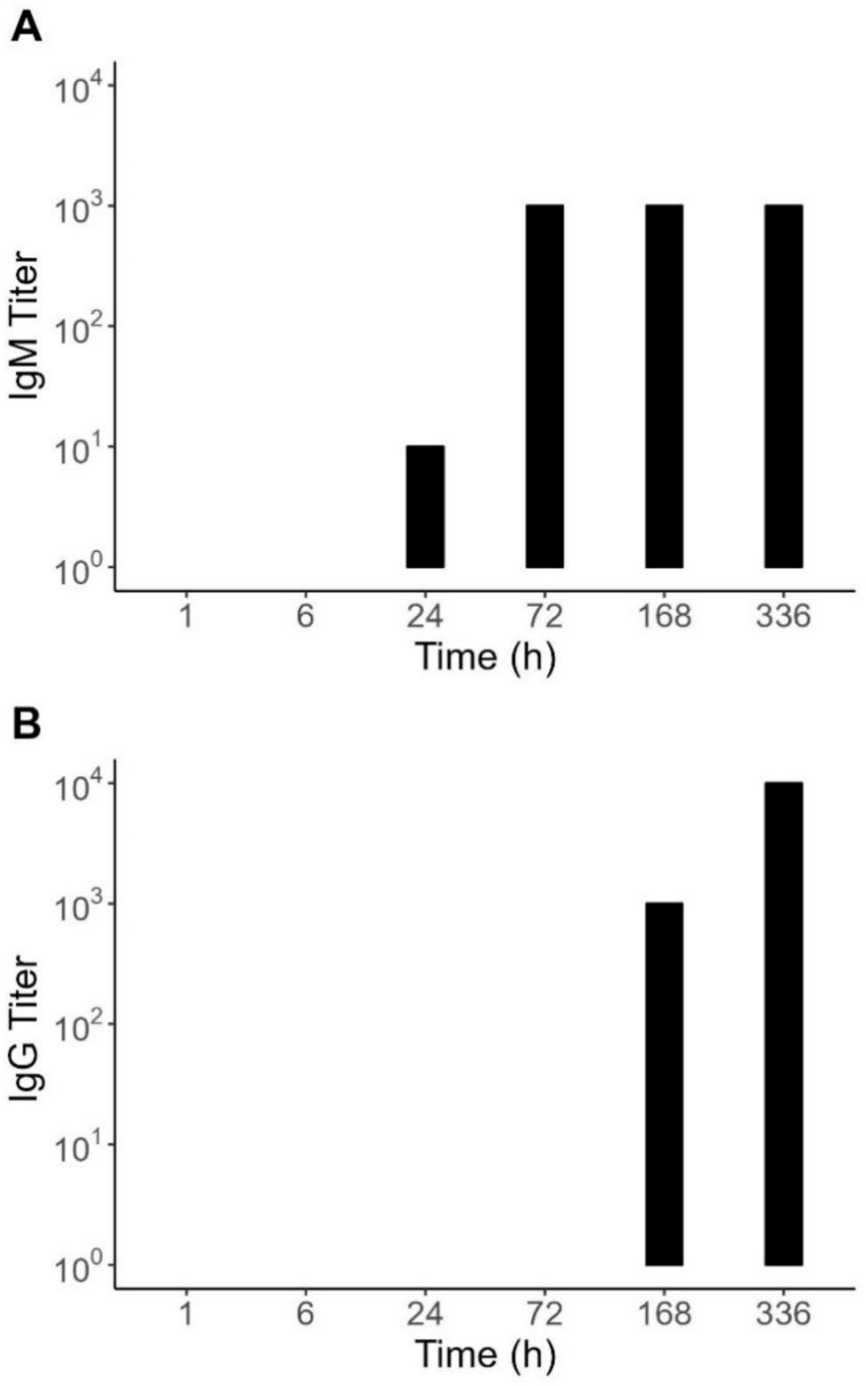
Temporal profile of anti-spike protein IgG and IgM antibody responses following mRNA-LNP administration. Serum samples were collected at defined time points and analyzed for spike-specific IgM and IgG levels. IgM response was detected early at 24 h, peaking at day 3 and persisted through day 14 following mRNA-LNP administration. The anti-spike IgG response developed later with first detectable titers on day 7, which further increased on day 14, reflecting progression of immune response.

Anti-spike protein IgM and IgG titers were measured in mice following a single 2 mg/kg intravenous dose of spike protein–encoding mRNA-LNP. Spike protein expression following mRNA LNP administration elicited a rapid and measurable humoral immune response. A clear temporal distinction between IgM and IgG responses was observed. Anti-spike IgM titers were first detectable at 24 h post-dosing at low titers (1:10) and increased markedly by 72 h (1:1000), remaining consistently elevated through 7 and 14 days (Fig. 7A). Anti-spike IgG was first detected much later on day 7 at a titer of 1:1000, indicating the initiation of class-switched antibody production. By day 14, IgG titers had further increased to 1:10,000 indicating continued expansion and affinity maturation of antigen-specific B cells (Fig. 7B). This pattern is consistent with the typical humoral immune response observed following SARS-CoV-2 infection or administration of immunogenic therapies, in which IgM antibodies appear first, followed by the emergence of IgG antibodies as the response matures [13, 15].

## Discussion

The mRNA-LNP platform has emerged as a promising tool for the treatment and prophylaxis of a wide range of diseases, including those previously considered untreatable [2]. Despite remarkable advances made in mRNA and LNP technology, it remains unclear how the distribution of lipid, mRNA and expressed protein relate to one another *in-vivo* [16]. To make informed formulation choices, it is critical to delineate the impact of LNP composition and mRNA design on mRNA delivery and downstream tissue specific protein expression. The selection of LNP composition for individual therapies can be guided by its tissue-specific mRNA delivery profile. Moreover, understanding tissue specific exposure of individual components (i.e., lipids, mRNA and expressed proteins) helps better understand potential toxicity of developed therapy. This is especially relevant for ionizable lipids as they are not endogenous to our body and can have long-term safety impact. Thus, in this study, we have quantified biodistribution of lipid, mRNA and expressed spike protein in plasma and tissues following spike protein expressing mRNA-LNP administration in mice. Since our objective was to gain a comprehensive understanding of whole body biodistribution of lipids and mRNA as well as tissue specific expression of encoded protein following mRNA-LNP administration, the biodistribution studies were conducted following intravenous dosing of LNPs.

Consistent with previous reports on lipid nanoparticles with diameters ranging from 90–200 nm, in our study, the highest levels of uptake were observed in the liver and spleen whereas the lowest uptake was seen in brain, followed by muscle [17]. While previous studies have reported similar nanoparticle biodistribution coefficients in liver and spleen for lipid nanoparticles encapsulating small molecules, our data indicates that ALC-0315 containing LNPs have almost fivefold higher uptake in the liver compared to spleen. This difference may be attributed to differences between the lipid composition of the LNPs used in these studies. Indeed, a study by Zhang et. al, found ALC-0315 LNPs to have greater hepatic tropism than other LNPs [18]. Lipid 5–containing mRNA LNPs also demonstrated preferential liver uptake, with approximately threefold higher ${C}_{max}$ in the liver compared to spleen, and the lowest uptake observed in the brain. Unlike ALC-0315 PK, which had similar terminal slope in all tissues, Lipid 5 exhibited tissue-specific pharmacokinetics, with a notably shorter half-life in the kidney, liver, and muscle, but prolonged retention in the spleen [10]. These results highlight the significant influence of LNP lipid composition on its tissue biodistribution and PK. Tissue distribution analysis also revealed significantly distinct ALC-0315 PK between plasma and other tissues in mice (Fig. 6A). While it is rapidly cleared from plasma with less than 1% of maximum plasma concentrations remaining at 24 h post dose, it persisted in tissues for several weeks, with > 10% of maximum tissue concentrations still present in liver and spleen 2 weeks post dosing. Our findings are consistent with observations of ALC-0315 concentrations in plasma and liver reported in rats post intravenous dosing of mRNA LNPs [19]. The distinct PK observed in plasma and tissues underscores the importance of evaluating lipid biodistribution in plasma as well as tissues.

Unlike other LNP components like cholesterol, phospholipid and PEG-lipids which are endogenous in nature, ionizable lipids are synthetically prepared. Studies have shown that ionizable lipids in mRNA LNPs such as ALC-0315, SM-102 can induce innate immunity and also elicit inflammatory response [20, 21]. In our study, we observed that ALC-0315 persists in tissues for long durations. While this may be acceptable and even advantageous in vaccine related applications which are administered intramuscularly and involve low, infrequent dosing meant to induce immunity, use of such lipids may pose safety concerns if applied for chronic therapies.

Encapsulated mRNA exhibited much faster clearance, with concentrations falling below detectable limits 168 h post dosing, which was distinct than ionizable lipid PK. While liver had the highest ALC-0315 exposure, maximum mRNA exposure was observed in the spleen. Although the mRNA uptake was higher in the liver at early time points, its concentration declined more rapidly compared to the spleen, resulting in the spleen exhibiting the highest mRNA levels at time points beyond 6 h. We hypothesize that this observation may stem from different LNP and mRNA turnover rates between liver and spleen tissues. Among the analyzed tissues, mRNA exposure was greatest in the spleen, followed by the liver, heart, kidney, and lung. These findings suggest that ALC-0315-containing LNPs may be particularly well-suited for therapeutic applications targeting the liver and spleen. In our study, the half-life of spike protein mRNA in blood was found to be 8.6 h, which is comparable to the reported half-life of mRNA (10.1–22.1 h) in other studies following mRNA-LNP administration in mice [22]. The divergent PK behavior of ALC-0315 and mRNA highlight that one component cannot serve as a surrogate for the other and it is essential to analyze PK of both individually.

In our study the highest concentrations of expressed protein were observed in the liver, which is similar to other published mRNA-LNP studies [6, 7, 10, 23]. Detection of spike protein in the liver as early as 10 min post-dose illustrates the remarkable speed of the entire process of LNP uptake, mRNA release, and protein translation, in contrast to other gene delivery platforms such as AAVs, which typically require several days for detectable protein expression [24]. Similar reports of protein translation as early as 10–15 min post mRNA-LNP dosing have been described in the literature [10, 25, 26], although most studies report detectable expression beginning 1 h post dose. This variability may reflect differences in experimental design, including later first sampling timepoints or limitations in the sensitivity of imaging studies used to detect protein concentrations. Interestingly, significant levels of spike protein expression were also detected in other tissues, particularly in the spleen, heart, kidney, and lung. A prior study with mRNA LNPs of similar lipid composition also found maximal protein expression in liver and spleen despite different administration routes [27]. This distribution pattern contrasts with that of Factor IX expression following administration of mRNA-LNPs formulated using ionizable lipid-Lipid 5, where protein levels in non-hepatic tissues were approximately 100-fold lower than those in the liver. The contrast in tissue expression profile suggests substantial impact of LNP lipid composition on tissue distribution of protein expression [10]. However, comparisons with Factor IX should be interpreted cautiously, as it is secreted, while spike protein is primarily membrane-bound.

Since we used the original viral signal peptide in our mRNA design (akin to Pfizer–BioNTech vaccine), spike protein is synthesized in the ER lumen and adopts its native membrane-bound form via S2 anchoring. Notably, without a mutated S1/S2 furin site, the spike protein may be cleaved and shed into circulation. Vesicular trafficking may also package some of the expressed protein into exosomes, contributing to its systemic presence [12]. In our study, minimum spike protein concentrations were observed in plasma 1 h post dosing. Concentrations increased at 6 h and remained stable until 72 h followed by elimination. By 168 h, highest spike protein concentrations were seen in plasma. While all examined tissues exhibited a similar elimination phase, plasma showed a distinctly slower elimination rate, indicating shedding from tissues at later timepoints. The rapid decline in spike protein concentrations after 72 h aligns with the observed onset of anti-spike IgM and IgG responses suggesting the potential role of immune mediated elimination. As such, our results suggest that systemic administration of mRNA-LNP can induce the immune response against the translated protein, which might be beneficial for vaccine development, but can be a liability for the development of therapeutic approaches.

Our study has several limitations which should be kept in mind as well. Animals in our PK studies were not perfused prior to tissue collection to minimize RNA degradation during tissue collection. Consequently lipid, mRNA and spike protein in residual blood within tissues will contribute to the measured tissue concentrations and must be accounted for when interpreting tissue specific distribution patterns. mRNA’s inherent susceptibility to degradation by RNases during tissue collection and processing may lead to incomplete extraction and underestimation of RNA levels. Although extraction efficiency was estimated using spiked samples for each tissue matrix, it was assumed to remain consistent across all samples from the same tissue type, a factor that may not hold true in practice. Furthermore, RT-qPCR is sensitive to inhibition by salts, residual contaminants, or even high total RNA concentrations, all of which can suppress amplification and yield artificially low values. Notably, previous studies have reported that RT-qPCR may underestimate RNA levels by 3–fourfold compared to the b-DNA assay, and this potential underestimation should be considered when interpreting our results [22, 28]. Finally, RT-qPCR and LCMS methods quantify total mRNA and ALC-0315 concentrations respectively, without resolution of LNP encapsulated and free forms. Thus, the pharmacokinetics of the ionizable lipid do not directly reflect those of the intact LNP, especially at later time points when LNP degradation results in the persistence of free ALC-0315 in the system.

In summary, this study offers a detailed quantitative evaluation of the biodistribution and PK/PD behavior of mRNA-LNP therapies post intravenous administration in mice. Our findings provide insights into the interplay between the different components of mRNA-LNP and reveal that tissues beyond the liver contribute substantially to protein expression. These results underscore the importance of evaluating the PK of mRNA LNP therapies not only in blood and liver, but across a broader range of tissues. The data generated here provides the foundation for the development of quantitative systems pharmacology (QSP) models for mRNA-LNPs going forward.

## Conclusion

In this manuscript we have presented quantitative whole-body biodistribution data ALC-0315 lipid, mRNA and expressed spike protein following intravenous administration of mRNA-LNP in mice. Our findings demonstrate that the PK of the lipid, mRNA, and expressed protein are markedly distinct, underscoring the need to evaluate all three components to fully understand mRNA-LNP pharmacology. The substantial differences between plasma and tissue kinetics indicate that plasma measurements cannot reliably serve as surrogates for tissue exposure. We also show that protein expression begins within minutes of dosing, highlighting the potential utility of mRNA LNPs in applications requiring rapid therapeutic onset. We also observed a robust humoral immune response against the expressed protein following systemic delivery of mRNA-LNP, which needs to be further validated across different formulations. The comprehensive PK data generated here provides impetus for developing QSP models for mRNA-LNPs.

## Supplementary Information

Below is the link to the electronic supplementary material.

Supplementary file1 (DOCX 204 KB)
