# Supplementary material for: Whole-Body Pharmacokinetics of Lipid, mRNA and Translated Protein Following Intravenous Administration of Spike Protein Expressing mRNA-LNP in Mice
Source: Pharm Res. 2026 Apr 2;43(5):1471–86. doi: 10.1007/s11095-026-04086-4 (PMC13269152; doi:10.1007/s11095-026-04086-4)
Supplement: Supplementary file 1 — Supplementary file1 (DOCX 204 KB) [file 11095_2026_4086_MOESM1_ESM.docx]

**SUPPLEMENTARY MATERIAL**

**Fig. S1.** Representative LC-MS/MS standard curve used for quantification.

**Fig. S2.** Representative RT-qPCR standard curve used for quantification.

**Fig. S3.** Representative ELISA standard curve used for quantification.

**Fig. S4.** Representative Ribogreen assay standard curve.


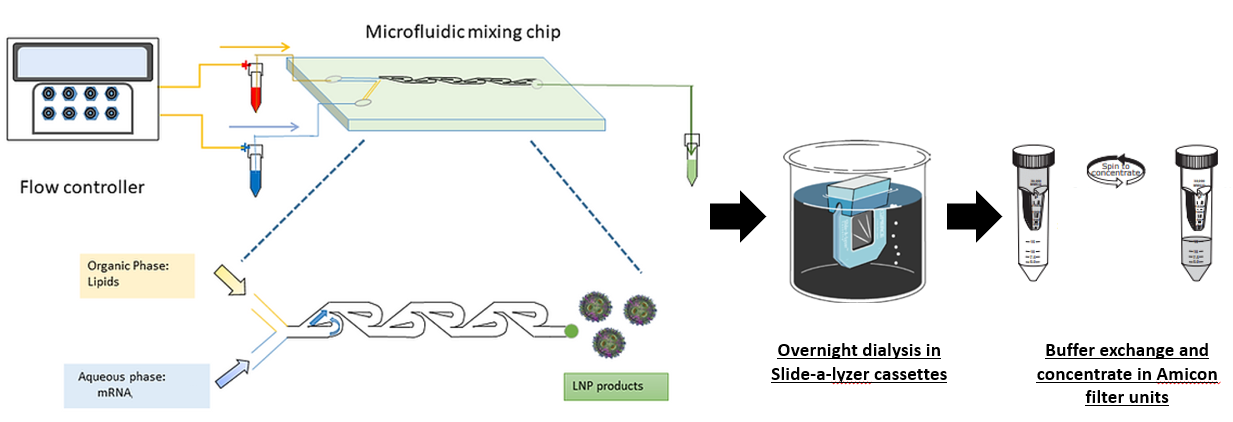


**Fig S5.** Schematic illustration of the mRNA-LNP production process with Flex-M machine (Precigenome).
